# Ten quick tips for computational analysis of medical images

**DOI:** 10.1371/journal.pcbi.1010778

**Published:** 2023-01-05

**Authors:** Davide Chicco, Rakesh Shiradkar

**Affiliations:** 1 Institute of Health Policy Management and Evaluation, University of Toronto, Toronto, Ontario, Canada; 2 Department of Biomedical Engineering, Emory University, Atlanta, Georgia, United States of America; SIB Swiss Institute of Bioinformatics, SWITZERLAND

## Abstract

Medical imaging is a great asset for modern medicine, since it allows physicians to spatially interrogate a disease site, resulting in precise intervention for diagnosis and treatment, and to observe particular aspect of patients’ conditions that otherwise would not be noticeable. Computational analysis of medical images, moreover, can allow the discovery of disease patterns and correlations among cohorts of patients with the same disease, thus suggesting common causes or providing useful information for better therapies and cures. Machine learning and deep learning applied to medical images, in particular, have produced new, unprecedented results that can pave the way to advanced frontiers of medical discoveries. While computational analysis of medical images has become easier, however, the possibility to make mistakes or generate inflated or misleading results has become easier, too, hindering reproducibility and deployment. In this article, we provide ten quick tips to perform computational analysis of medical images avoiding common mistakes and pitfalls that we noticed in multiple studies in the past. We believe our ten guidelines, if taken into practice, can help the computational–medical imaging community to perform better scientific research that eventually can have a positive impact on the lives of patients worldwide.

## Introduction

Medical images are useful data elements that provide visual and spatial information regarding the condition of a particular organ or tissue of a patient. Medical images supply a representation of a health condition to medical doctors, who can interpret them and made better decisions regarding treatments or therapies. Since the 1970s, new biomedical engineering technologies have allowed the medical community to develop novel medical imaging modalities to evaluate and assess the medical conditions of patients. To mention a few, these image modalities include X-ray radiography, magnetic resonance imaging (MRI), positron emission tomography (PET), computed tomography (CT) scan, and single-photon emission computed tomography (SPECT). Since 2010s, new computational analyses of medical images have been made possible through machine learning [1–3]. In the meanwhile, open access medical images have become available online on several data repositories. While performing computational analyses of medical images has become easier, making mistakes during these analyses has become easier, too.

Following the examples of the debates about best practices for machine learning in bioinformatics [4–9], best practices for machine learning in health informatics [10–12], best practices for computational statistics [13,14], and best practices for pathway enrichment analysis [15–17], we decided to present here ten quick tips to perform computational analyses of medical images avoiding common mistakes and pitfalls that we noticed in multiple studies on this topic. In the biomedical literature, some studies already reported guidelines for machine learning applications to medical image analysis [18–21]: Most of those articles, although useful, are limited to computational intelligence.

With this article, instead, we propose ten quick tips not only related to data mining and pattern recognition, but to any computational analysis of health imaging data, including segmentation, coregistration, quality improvement, and classification. In the past, the PLOS Computational Biology Quick Tips series published no articles on guidelines for medical images and released only a study on scripts for neuroimaging [22]; we fill the gap by presenting this study. We designed these simple recommendations for beginners, but we believe they should be kept in mind by experts, too.

### Tip 1: Before starting, make sure you have enough computational resources

As simple as it might seem, before starting any computational analysis, you have to make sure you have enough computational resources, both in terms of hard disk drives and random-access memory (RAM) slots. For other biomedical informatics fields, such as bioinformatics or analysis or electronic health records, one might tend to forget about this issue, because the data files are rarely so large to need special treatments.

A recent study one of us authors published, for example, involved the machine learning analysis of data of patients with inflammatory bowel disease [23]: The dataset analyzed was contained in a comma-separated values (CSV) file of 13 kilobytes size, previously released openly on FigShare [24]. Another study involved the usage of microarray gene expression data of patients with heart failure [25], whose dataset was originally released on Gene Expression Omnibus (GEO) as a TAR archive of CEL files of 1.8 gigabytes [26]. For the computational analysis done on both these datasets, a personal computer with limited resources such as Dell Latitude E5420 with Intel Core i5-2520M central processing unit (CPU) @ 2.50 gigahertz processor and 5.7 gigabytes RAM was sufficient, but it would be unsuitable for computational analyses of larger datasets, such as medical images.

Medical image datasets, in fact, have much larger sizes. The collection of magnetic resonance imaging scans for glioblastoma patients from the University of Pennsylvania Health System on the Cancer Imaging Archive (TCIA) [27], for example, contains Digital Imaging and Communications in Medicine (DICOM) files of 139.4 gigabytes. And the dataset of the OASIS brain project of neuroimaging scans weights approximately 114 gigabytes [28,29], just to name another one. With datasets of these sizes, most common laptop would be insufficient to perform any scientific analysis in reasonable time. To be more precise, not only medical images’ data require larger computational resources, but also bioinformatics data (such as single-cell RNA-seq [30], for example) could easily need more than hundreds of gigabytes of disk spaces, when thousands or millions of cells are involved in the studies.

Scientific analysis of large datasets like single-cell RNA-seq data or medical images necessitates of high performance computing systems able to process a fair number of medical images in few minutes [31,32]. Moreover, computational resource allocation depends also on which computational techniques you plan to use for your scientific analysis: deep learning models (for example, convolutional neural networks; [33]) would require more RAM memory and disk space than simpler machine learning methods (such as linear regression) or traditional statistics tests (for example, Mann–Whitney U test; [34]), of course. So, in addition to the data size, you need to consider which methods you would like to employ.

Therefore, here is our first simple tip: Before embarking in an image analysis project, make sure you enough computational resources to perform the analysis. If necessary, go and talk to the computational resources department of your institute or company. Usage of high-performance computing (HPC) [35] or graphics processing units (GPUs) [36] can be beneficial for the analysis.

### Tip 2: Before starting, make sure you have enough medical images for your study

Presentation of disease patterns on medical imaging is often subtle, and radiologists require significant training to identify them. Use of machine learning algorithms to automate the task of detection and classification of disease patterns on medical imaging is often difficult. Therefore, a sufficient number of studies that capture different presentations of disease patterns under varying conditions including scanner differences, extent of disease, and subtypes of disease is required to train reliable and reproducible machine learning models.

Unfortunately, a number of machine learning studies that have been published in the past were just proofs of concept and preliminary studies. They have often used small datasets and have demonstrated the promise of the machine learning potential; however, such studies have often failed when validated on external datasets [37]. As the machine learning field has matured, one of the important considerations for publishing high-impact studies is to demonstrate the reproducibility of these methods [38].

While we cannot provide a general rule of thumb for the sample size required for given study, we recommend that power analysis and sample size calculations are conducted prior to conducting experiments. Several methods and software programs are available for sample size calculations such as MedCalc [39], Power Analysis and Sample Size (PASS) [40], R sampler and pwr packages [41,42], and IBM Statistical Package for the Social Sciences (SPSS) [43], which can help determine sample size for a given study.

Another aspect to keep in mind for binary classification and regression analysis tasks is class imbalance: When dataset has skewed class proportions, with one class being much larger than the others, some problems may arise, which might compromise the prediction task [5]. In these cases, we advise using techniques for class imbalance data handling [44], such as data weighting [45], oversampling [46], undersampling [44], and data augmentation [47]. The data imbalance issue, moreover, can be noticed and handled only by using metrics that take it into account, such as the Matthews correlation coefficient (MCC) for binary classifications and the coefficient of determination (R^2^) for regression analyses (Tip #8).

### Tip 3: Take care of noise and apply image harmonization

#### Noise

Inexperienced users and beginners often do not know about it, but medical images always come with noise. Always. Noise is an invisible issue: You might think it is not there, but it is. And repositories, which provide medical images, do not mention it, because it is implied: No repository presents their data by writing something like “breast cancer screening mammograms images with noise,” for example.

The bad news is that noise is there, anyway. The good news is there are methods for handling them, called denoising techniques [48]. Specific denoising techniques are suitable for specific medical images, such as X-ray radiography images [49–51], CT scan images [52–54], magnetic resonance images (MRI) [55–57], ultrasound images [58,59], positron emission tomography (PET) images [60], just to mention a few. Our recommendation, simply, is not to analyze the raw dataset as it comes, but always remember to perform a denoising step first.

#### Harmonization

Data harmonization implies minimizing biases and variations that might arise on account of differences in scan parameters, post-processing protocol, and site-specific aspects [61–63].

When datasets from multiple institutions and hospitals are involved, harmonization becomes crucial to ensure that studies belonging to a specific class have similar intensity ranges and patterns. There are several open source tools available that can assist in checking the presence of batch effects arising due to scanner and site-specific variations. MRQy [64] and LAB-QA2GO [65], for example, are some recently developed tools with the aim of identifying batch effects and assist in quality control. There are several methods available for data harmonization.

One of the simplest methods is histogram matching, where intensity distributions of scans from multiple batches (scanners/sites) are aligned, rendering the processed scans to share a similar appearance [66]. Some of the more recent and advanced techniques use deep learning for harmonizing datasets [67]. In certain cases, despite the use of image harmonization tools, batch effects continue to persist and in such cases, one might make a call to develop and validate machine learning or computational image analytic approaches that are specific to a particular site or scanner.

Additionally, we can consider using computational imaging approaches that are independent of image intensity measurements such as shape and volume based features [68].

### Tip 4: Only use open source programming languages and software platforms

When one decides to start a project involving computational analyses of medical images, they face an important question: Which programming language and software platforms should I use for this study? The answer is clear and straight for us: a fast, open source one like Python, R, C, C++, or Java.

Using an open source programming language brings several benefits and advantages. First, the programmer would be able to easily and freely share their software code with collaborators, and even reuse it after the project has finished, even if they moved to a different institution or company. Moreover, using an open permissive software license would allow the reproducibility of the computational analysis [69]. We also suggest to keep an eye on the TIOBE Programming Community Index [70] to get updates on the most used programming languages worldwide.

Since Python is not only the most popular programming language in machine learning and data science, but also the most used programming language in any field [70], we recommend all the beginners and students to start their projects with it. Moreover, the software packages’ catalogue of Python include some popular, effective software libraries for deep learning, such as TensorFlow [71] and PyTorch [72].

Avoid proprietary software: Proprietary software programs cannot be shared openly and restrict their usage only to users who have their expensive licenses. Moreover, what is the point of spending your money or your institution’s money for a license when free, open source alternatives are available? Scientific researchers often work in public universities and research centres funded by taxpayers’ money. In this context, we suggest to keep in mind the quote of the economist Bruno Leoni: “Money of everybody should not be treated like money of nobody.” At the end of the project, we also advise releasing publicly your software code on public repositories such as GitLab or GitHub, to enhance reproducibility [69], and facilitate the detection of possible mistakes in the analyses [73].

### Tip 5: Identify features specific to your scientific problem

Computationally derived features provide alternate representations that enhance specific attributes and patterns for specific tasks. For instance, feature representations have been used for coregistration [74], segmentation [75], and classification [68].

Tasks using medical imaging data typically take advantage of handcrafted features from the diseased regions of interest to train on. Pyradiomics is a popular open source radiomic package that can serve as a general start to extract computational features from radiology imaging data [76]. This library includes intensity statistics, first order, second order, co-occurrence-based features. This can be a starting point for several classification tasks where we would like to quantify lesion characteristics using quantitative features.

However, depending on the clinical problem and disease, the starting set of features can be different. For instance, if we are planning to quantify the shape of a lesion, then volume and shape-based features such as fractals [77]. In case, lesion edge and gradients are the region where differential characteristics of less and more aggressive lesions are observed, then we might start off with edge-based features [78]. As deep learning methods are becoming popular, they are also being used as feature extractors [79]. Features from deep learning networks can be integrated with other clinical features, which may not be possible when using end-to-end deep learning networks.

Deep learning features can also be used in conjunction with simpler machine learning classifiers such as Naïve Bayes or logistic regression. These pieces of advice are useful not only for machine learning but also for segmentation [80] and coregistration [81] tasks.

### Tip 6: Reduce overfitting through feature selection and dimensionality reduction

Classification is one of the most common tasks in computational imaging, and one of its important challenges is to avoid overfitting for the models to be generalizable and reproducible [82]. As mentioned earlier, open source feature extraction libraries such as Pyradiomics [76] provide a large initial set of features.

When these techniques are used to train machine learning models, overfitting occurs, and this problem is commonly known as the curse of dimensionality [83]. To avoid this problem, we need to reduce the set of features we work with. As a rule of thumb, use less than 10% of the sample size as the number of features for classification problem. For machine learning and deep learning tasks, imaging data are first converted to a feature vectors on which feature selection and dimensionality reduction techniques are then employed.

The feature vector usually includes shape features, color histogram features, color moment features, and texture features [79]. Often the success of a machine learning application depends on how accurately one can quantify input data (image or volume or text) in terms of quantitative features. To get to a reduced feature set, there are basically two methods one can adopt: feature pruning and dimensionality reduction.

#### Feature pruning

Feature pruning approaches involve discarding those features that are not important for the classification problem.

One way to approach this is to identify features that show significant differences between different classes using a *t* test and only retain those. Another way to do this is to remove features that are highly correlated with each other and essentially are duplicates of each other. Correlation coefficients such as Pearson’s correlation coefficient (PCC) can be used to set threshold (for example, PCC > 0.9) and discard features that are highly correlated. Lastly, feature selection methods (for example, minimum redundancy maximum relevance (MRMR); [84]) and feature ranking methods (for example, feature importance in nonlinear embedding (FINE); [85]) can be used to identify a subset of features that can be used for machine learning.

#### Dimensionality reduction

Dimensionality reduction approaches essentially identify characteristics of the features in the high dimensional space and determine approximations in the low dimensional space [86,87].

A popular dimensionality reduction method is principal component analysis where eigenvectors pertaining to the top eigenvalues are used to obtain feature characteristics [88]. Machine learning models can then be trained on these low dimensional feature vectors. Recently, embedding approaches are being widely used for dimensionality reduction.

Uniform manifold approximation and projection (UMAP) [89] and t-distributed stochastic neighbor embedding (t-SNE) [90] are popular embedding approaches for visualization used in a wide variety of machine learning applications. Choice of feature selection and dimensionality reduction depends on whether one cares about the interpretability of the features. Feature selection retains the original features that can then be overlaid over medical imaging data on a pixel-wise basis for visualization and interpretation.

However, if all one cares is a good classification performance, dimensionality reduction methods provide an efficient means.

### Tip 7: Split your dataset into training set and test set, and look for a validation cohort dataset online

#### Dataset split

Splitting the dataset into a training set and a test set, by making sure that no data element is shared between the two, is a key pillar of any supervised machine learning analysis [5]. We would like to reaffirm this concept and enforce it by stating clearly that the same practice should be employed also in any studies, even the ones not involving a computational intelligence phase.

Even for probabilistic projects or unsupervised machine learning studies, in fact, splitting the dataset into two different subsets is a good idea for validation purposes. In a cluster analysis applied to medical images, for example, one can split the dataset into 60% randomly selected images for the first set and using the remaining 40% for the second set, applying the computational method to the former one, then to the latter one, and comparing their results. If the researcher observes the same scientific results on both the subsets, they can consider the results more consistent.

Of course, this split should not be performed only once: The researcher should repeat the same analysis at least 100 times (if they have the sufficient computational resources, as explained in Tip #1), by splitting the dataset in different ways each time. If, at the end of 100 executions, a scientific result always shows up on both the subsets, it can be considered truthful.

#### Validation cohort search

A key component of solid scientific studies is the repetition of the application of same method on a second dataset, with the same features and a different origin, usually called validation cohort. Often, however, it is difficult to find an alternative dataset where to repeat a computational analysis.

Even if it is difficult to find the right resource, we suggest the readership to look for an alternative dataset on public web repositories of image data such as the Cancer Imaging Archive (TCIA) [27], Open Access Series of Imaging Studies (OASIS) [28,29], and OpenfMRI [91,92], or on general data repositories such as Re3data [93], Google Datasets Search [94], Kaggle [95], and the University of California Irvine Machine Learning Repository [96].

We know that a bit of luck is fundamental to find a dataset having the same disease and of the same features as the one used in the primary analysis, but it never hurts to try. Of course, obtaining the same results on a different dataset by using the same method employed on the primary dataset would make the study much more robust and valid.

### Tip 8: Use multiple evaluation metrics to assess your results, not only one

A common mistake we see in multiple health informatics studies is reporting the results measured with only one rate that usually is the most common rate of the scientific field. This approach has a hidden problem: Since no statistical rate is perfect, the results measured only with one single rate are going to hide some drawbacks and bad news. For this reason, we recommend anyone performing any analysis of medical images to employ multiple statistical rates, and not only one.

A study whose results are based only on receiver operating characteristic (ROC) area under the curve (AUC), for example, would hide the performance of the binary classification measured as precision (PPV = TP/(TP + FP)) and negative predictive value (NPV = TN/(TN + FN)), where TP are true positives, FP are false positives, TN are true negatives, and FN are false negatives.

For binary classifications, we suggest to always include at least the MCC [97], sensitivity, specificity, precision, and negative predictive value, and to base the rankings of the methods on the MCC [98–102]. Never base all your results on ROC AUC [103–107]. ROC AUC, accuracy, F1 score, and most common statistical rates, in fact, might illude clinicians and researchers when analyzing classification dataset with skewed class proportions. The MCC, on the other hand, is the only statistic that generates an informative, truthful outcome for binary classifications [98–102]. Regarding regression analyses, we recommend to always include at least coefficient of determination (R-squared or R^2^), symmetric mean absolute percentage error (SMAPE), mean square error (MSE), root mean square error (RMSE), mean absolute error (MAE), mean absolute percentage error (MAPE), and build the rankings of the methods on R-squared [108].

For clustering analysis, always include Davies–Bouldin index [109], Dunn index [110], and silhouette coefficient [111]. Depending on your scientific goal, considering results measured through mutual information, adjusted mutual information, Rand index, adjusted Rand index, homogeneity, completeness, and V-measure could be a good idea. When doing statistical and probabilistic analyses, always include at least adjusted *p*-value, nominal *p*-value, *q*-value, and *z*-score, if available, and rank the methods based on the former one [112].

Of course, particular statistics can be utilized based on the scientific goals of a study (for three-dimensional medical image segmentation [113], for example), but we would like to reaffirm our take-home message here: Never use a singular rate, always employ multiple statistics.

### Tip 9: Ask a radiologist or a medical doctor to review your medical image analysis results

After months of work, multiple executions of your software, your analysis is done, and you are collecting the results, making them ready for the manuscript. You feel like you did your homework, you reached the mountaintop, and now you just need to present your methods and results in an article draft. Before writing your paper, however, there is a final step of your analysis that we suggest you to do: Show your results to a radiologist or a medical doctor, and ask them their honest feedback about them.

Are your results something they would expect to see for patients of that particular disease? Do the results make sense? How do your medical results relate to the existing knowledge in the biomedical literature? These are all pertinent questions you might ask your medical collaborator. Their external, agnostic feedback would be pivotal for your study: If they approved your results, you would rest assured about the quality of your analysis; otherwise, if they noticed something wrong, it would be much better to hear the bad news from them informally and confidentially before submitting the article than having your article rejected by a scientific journal later on.

Additionally, when analyzing patients’ data with computational intelligence, researchers should investigate the interpretability of their statistical models, by opening the black box to explain what the model does. Frameworks for explainable artificial intelligence (XAI) can be employed [114]. Van der Velden and colleagues [115] write about interpretable AI: “Something can be considered a good explanation if it gives insight into how a neural network came to its decision and/or can make the decision understandable.”

Interpretation of the behaviors of machine learning models applied to medical images can then be assessed through an application-grounded evaluation, where a domain expert can inspect the behavior of the machine learning model, compare the actual results with their expected results, and eventually say if the interpretation is correct [115]. XAI is a broad theme and its discussion goes beyond the scope of this study; however, we advise the readership considering the importance of interpretability of computational intelligence models applied to medical images [18,116–120].

### Tip 10: If possible, release your dataset publicly online

Earlier, we recommended to look for validation cohort datasets on public online repositories (Tip #7). Here, we follow up on that piece of advice to giving back to the online communities rather than only receiving: After finishing your medical image analysis, if you are authorized, we recommend you to publicly share online your dataset on repositories such as Kaggle [95], the University of California Irvine Machine Learning Repository [96], FigShare [121], or Zenodo [122], following the findability, accessibility, interoperability, and reusability (FAIR) principles [123].

Of course, to do so, you first need to verify you have all the authorizations from the ethical committee of your institutions or hospital and the authorizations from the patients or the patients’ families to use their data. If you have them, releasing your dataset online can actually bring multiple advantages for your study.

First of all, it would allow the reproducibility of your analysis: Anyone around the world would be able to repeat your analysis and reach the same conclusions. Moreover, they would be able to find any potential mistakes, which is a fundamental aspect of scientific research involving patients. Additionally, users around the world would be able to reuse your data and generate different, additional results, building on your original outcomes. This aspect would also increase the impact of your study, in terms of article citations and in other ways.

If you also have the chance to decide on which journal to submit your article, we advocate to select an open access one: If published, your article will be readable by way more people in the world than if it was published in a restricted-access journal. An updated list of open access journals of health informatics can be found on Scimago Journal Ranking [124].

## Conclusions

Medical imaging is an efficient and useful tool that can show particular aspects of patients’ organs and tissues that would otherwise would be unnoticeable with traditional medical techniques. Through medical images, physicians can infer new information about the conditions of a patient and, therefore, design a better therapy.

Computational analysis of medical images can highlight particular trends among those images, indicating some correlations between patients that otherwise would be unobservable. Looking at images does not only allow the observer to see specific conditions; more importantly, it facilitates the understanding of a phenomenon.

As the computer vision expert Tomaso Poggio once wrote [125], it is not by chance that in the English language people use the term “I see” as a synonym of “I understand.” While computational tools for medical image analysis have become more common, making mistakes have become easier as well. With these quick tips, we report a list of best practices for computational analyses of medical images that can help users avoid common mistakes and wrongdoings. We believe our ten recommendations can help researchers generate better and more reliable results, ultimately leading to better treatments and cures for patients.
